# 17-Hydroxyprogesterone caproate to prolong pregnancy after preterm rupture of the membranes: early termination of a double-blind, randomized clinical trial

**DOI:** 10.1186/1756-0500-4-568

**Published:** 2011-12-29

**Authors:** C Andrew Combs, Thomas J Garite, Kimberly Maurel, Kimberly Mallory, Rodney K Edwards, George Lu, Richard Porreco, Anita Das

**Affiliations:** 1 Obstetrix Medical Group, Center for Research, Quality, and Education, San Jose, CA, USA; 2 Obstetrix Medical Group, Center for Research, Quality, and Education, Steamboat Springs, CO, USA; 3 Obstetrix Medical Group, Center for Research, Quality, and Education, Fountain Valley, CA, USA; 4 Obstetrix Medical Group, Phoenix Perinatal Associates, Phoenix, AZ, USA; 5 Obstetrix Medical Group of Kansas City, Kansas City, MO, USA; 6 Obstetrix Medical Group of Colorado, Denver, CO, USA; 7 AxiStat, Inc., San Francisco, CA, USA; 8 Obstetrix Medical Group, 900 E Hamilton Avenue #220, Campbell, CA, USA

## Abstract

**Background:**

Progestational agents may reduce the risk of preterm birth in women with various risk factors. We sought to test the hypothesis that a weekly dose of 17-hydroxyprogesterone caproate (17P) given to women with preterm rupture of the membranes (PROM) will prolong pregnancy and thereby reduce neonatal morbidity.

**Methods:**

Double-blind, placebo-controlled randomized clinical trial. Women with PROM at 23.0 to 31.9 weeks of gestation were randomly assigned to receive a weekly intramuscular injection of 17P (250 mg in 1 mL castor oil) or placebo (1 mL castor oil). The primary outcome was the rate of continuing the pregnancy until 34.0 weeks of gestation or until documentation of fetal lung maturity at 32.0 to 33.9 weeks of gestation. Planned secondary outcomes were duration of latency period and rate of composite neonatal morbidity. Enrollment of 111 participants per group, 222 total, was planned to yield 80% power to detect an increase in the primary outcome from 30% with placebo to 50% with 17P.

**Results:**

Twelve women were enrolled of whom 4 were randomly assigned to receive 17P and 8 to receive placebo. The trial was terminated prematurely because of two separate issues related to the supply of 17P. No adverse events attributable to 17P were identified.

**Conclusion:**

Because of premature termination, the trial does not have adequate statistical power to evaluate efficacy or safety of 17P in women with PROM. Nonetheless, ethical principles dictate that we report the results, which may contribute to possible future metaanalyses and systematic reviews.

**Trial Registration:**

ClinicalTrials.gov: NCT01119963

Supported by a research grant from the Center for Research, Education, and Quality, Pediatrix Medical Group, Sunrise, FL

## Background

Preterm rupture of the membranes (PROM) occurs in 2-4% of singleton pregnancies, yet accounts for 18-20% of perinatal mortality in the United States [1]. PROM is frequently followed by preterm birth (PTB) within a few days [1,2]. Management of PROM involves a weighing of risks of prematurity complications versus risks of prolonging the pregnancy, such as chorioamnionitis, placental abruption, or cord prolapse. With PROM before 32 weeks of gestation, the morbidity of PTB is high, so attempts to prolong the pregnancy are generally advocated [1,2], a strategy called "expectant management." With PROM after 34 weeks, or after 32 weeks if fetal lung maturity is documented, the complications of PTB are less and the risks of infection and other complications of expectant management remain, so delivery is generally recommended [1,2].

Attempts to prolong the pregnancy with expectant management of PROM are often unsuccessful. The rate of delivery during expectant management of PROM roughly follows a "half-life" function, with about half of patients delivering within a week and about half of the remainder delivering with each subsequent week [3-6]. Antibiotic prophylaxis after PROM has been shown to prolong the latency period [3-7] (interval from PROM-to-delivery) and reduce neonatal morbidity [6,7]. Tocolysis has not been shown to be effective after PROM [8,9].

Progestational agents such as progesterone and 17-hydroxyprogesterone caproate (17P) have been shown to reduce the rate of PTB in women with certain risk factors for PTB, including women with a history of a prior PTB [10-12], women with a short cervix [13-15], or women who have had an acute episode of preterm labor successfully suppressed after tocolysis [16,17]. It is unknown whether 17P or other progestins might be beneficial after PROM. Progestins have multiple actions that might potentially help to prolong pregnancy after PROM, including suppression of inflammatory mediators and decreased production of contraction-associated proteins such as gap junction proteins and oxytocin receptors [18]. Briery and colleagues reported a small trial that showed no benefit of 17P versus placebo after PROM [19], but only 35 subjects had actually received 17P, so statistical power to reach a negative conclusion was rather limited.

The present study was undertaken to test the hypothesis that 17P given to mothers with PROM before 32 weeks of gestation will prolong the pregnancy and thereby reduce neonatal morbidity.

## Methods

The trial was sponsored, designed, and conducted by the Obstetrix Collaborative Research Network, a consortium of maternal-fetal medicine practices across the United States. The trial was approved by the independent Institutional Review Board (IRB) at each site. The study was conducted under Investigational New Drug (IND) Number 107785 through the United States Food and Drug Administration (FDA). The trial was registered on http://clinicaltrials.gov, #NCT01119963. An independent Data and Safety Monitoring Board (DSMB) supervised the trial, reviewed adverse event reports, and approved the premature termination of the trial. Additional File 1 contains the complete, IRB-approved, final protocol.

Women were eligible if they were at least 18 years old, had a singleton pregnancy at 23.0 to 31.9 weeks of gestation, and PROM. PROM was defined as either (a) documentation of vaginal leakage of indigo carmine dye instilled via amniocentesis; (b) a positive Amnisure^® ^test (Amnisure International, Cambridge, MA); or (c) two or more of the following: nitrazine test of vaginal secretions with pH 7 or higher, ferning of vaginal secretions, gross pooling of clear fluid in the posterior vaginal fornix, or ultrasound exam showing oligohydramnios. We excluded women with active preterm labor, defined as 8 or more uterine contractions per hour that were perceived by the patient and/or a cervical dilation 4 cm or more. The definition of PROM did not require that the rupture of membranes had occurred before the onset of labor (commonly called "premature" or "prelabor" PROM); that is, women were eligible if they had labored before PROM provided that they were not in active labor, as defined, at the time of enrollment. We excluded women with contraindications to expectant management (such as suspected intraamniotic infection, nonreassuring fetal heart rate tracing, fetal death, preeclampsia, active uterine bleeding, or documented fetal lung maturity), with known fetal abnormalities (such as major congenital malformation, viral infection, or hydrops), with history of allergy to 17P or castor oil, with medical conditions that might adversely interact with 17P (such as asthma requiring medications, renal insufficiency, seizure disorder, ischemic heart disease, cholecystitis, impaired liver function, or history of venous thromboembolism, breast cancer or depression requiring hospitalization), with medical conditions treated with systemic steroid medications, or with a cervical cerclage present at the time of PROM. Eligible women were approached by a physician or research nurse and were offered participation in the trial.

Women who gave informed consent were randomly assigned in a 1:1 ratio to receive either 17P (250 mg in castor oil, 1 mL total volume, intramuscular injection weekly) or an identical-appearing placebo (1 mL castor oil only). A computer-generated random-number sequence was used by the trial statistician to generate a randomization code book kept at each site's inpatient pharmacy. Study medications (17P versus placebo) were prepared by McGuff Pharmaceuticals (Newport, California) according to the IND specifications, which followed current Good Manufacturing Practices (cGMP). Medications were provided in 5 mL multi-dose vials, specially labeled for the study and number-coded to correspond with the randomization code book. Randomization was stratified by gestational age, 23.0-25.9 weeks, 26.0 to 28.9 weeks, and 29.0 to 31.9 weeks. Participants and research personnel were blinded to group assignment throughout, from before enrollment until after completion of all case report forms and resolution of all data queries. The code was not broken until after the database was "locked."

Each week from randomization until delivery, the assigned medication (17P or placebo) was drawn into a syringe labeled "Study medication: progesterone or placebo" by the inpatient pharmacy at each site, delivered to the nursing unit, and administered by a registered nurse. In the syringes, 17P and placebo appeared visually identical.

Other than the administration of study medication (17P or placebo), the remainder of each patient's clinical care followed standard clinical management for PROM. The investigators agreed in advance on many items of standard management. These included inpatient hospitalization of all patients until delivery, administration of a single course of antenatal corticosteroids, broad-spectrum antibiotic prophylaxis for 1 week, avoidance of tocolytics (except during the 48 h after the first dose of corticosteroids), and fetal heart rate monitoring at least 1 h daily. We encouraged amniocentesis to rule-out intraamniotic infection before enrollment, but did not require this. We discouraged digital examination of the cervix, but did not exclude subjects who had already had digital examinations. The protocol called for collection of amniotic fluid from vaginal leakage at 32.0 weeks or beyond for assessment of fetal lung maturity according to whatever test was in use at the local site. Intrapartum antibiotic prophylaxis (e.g. group B streptococcus coverage) was encouraged but not mandated. A protocol was in place for evaluation and management of suspected membrane resealing, but this was not used because all the participants continued leaking amniotic fluid until delivery. Management decisions not specifically addressed by protocol were left to the discretion of the managing physician, including route of delivery, choice of antibiotics, and other matters.

The primary outcome was defined as prolongation of the pregnancy until a favorable gestational age, which we defined as either 34.0 weeks of gestation or documentation of fetal lung maturity at 32.0 to 33.9 weeks. The investigators agreed that continuation of pregnancy beyond these time points was not indicated. Secondary outcomes were latency (interval from randomization to delivery) and composite neonatal morbidity, which was defined per Mercer et al. [6] as one or more of: stillbirth, neonatal death, infant death before hospital discharge, respiratory distress syndrome (RDS), intracranial hemorrhage (ICH) grade 3 or 4, necrotizing enterocolitis (NEC) stage 2 or 3, culture-proven neonatal sepsis within 72 h of birth. In addition to the components defined by Mercer et al. [6], our definition of composite neonatal morbidity included periventricular leukomalacia (characteristic lesions in the subcortical white matter seen on cerebral imaging studies within 96 h of birth.)

Sample size calculations indicated that we would need 105 participants in each group (17P or placebo) to yield 80% power to detect a 20% absolute increase in the rate of the primary outcome from 30% in the placebo group to 50% in the 17P group. This was adjusted to 111 per group to account for a possible 5% rate of loss-to-follow-up due to membrane resealing or other factors. A single interim analysis was planned when data were available for 50% of the total sample, but this was not performed owing to early termination of the study. Because of early termination, the trial is grossly underpowered; therefore, we present only descriptive statistics by treatment group and we did not perform any formal statistical tests of potential between-group differences.

The trial was designed, conducted, analyzed, and reported according to the principles outlined in the CONSORT Statement and Checklist [20].

## Results

**Figure 1:**
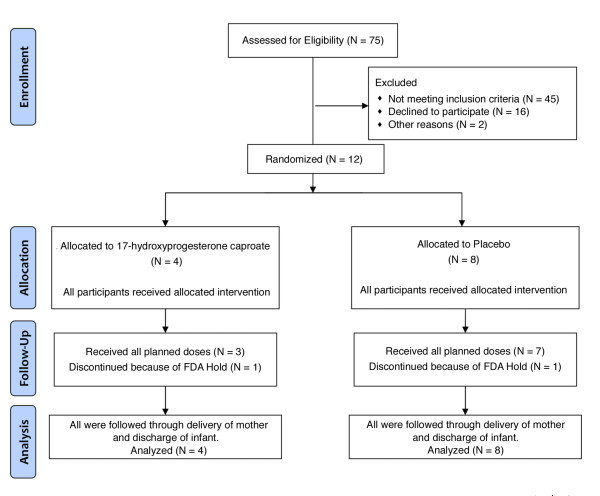
**Flow diagram showing number of participants at each stage of trial**.

From October, 2010 through January, 2011, twelve participants were enrolled, of whom 4 were randomly assigned to receive 17P and 8 were assigned to receive placebo. Figure 1 is a flow diagram showing the number of women at each stage of the trial.

On December 28, 2010, the FDA sent a Warning Letter [21] to McGuff Pharmaceuticals citing certain violations of cGMP in the company's manufacturing facility. Officials at McGuff disclosed this letter to us, stating that it did not relate to the production of 17P or placebo for our trial because the study medications were prepared in a segregated facility, not the general manufacturing line of the company cited in the Warning Letter. McGuff had sent periodic aliquots to an independent testing laboratory to verify the potency, sterility, and stability of the preparations throughout this trial and our previous trials evaluating 17P in twin [22] and triplet [23] pregnancies. Despite these reassurances, our DSMB recommended that we seek specific guidance from the FDA as to whether the Warning Letter impacted our trial. The FDA Division of Reproductive and Urologic Drug Products asked for copies of the documentation of the independent testing. Despite extensive documentation that the samples were sterile, pure, and stable, the FDA placed the trial on "Full Clinical Hold" on January 24, 2011. At that time, two women were still receiving study medications and we immediately discontinued any further administration of the drugs to them. We stopped all further recruitment into the study and notified all the local-site IRBs and the DSMB. We returned all the study medications to McGuff to be quarantined. We had planned to petition the FDA to remove the clinical hold and then to resume enrollment after McGuff had satisfactorily addressed the concerns outlined in the Warning Letter.

On February 4, 2011, the FDA granted approval to KV Pharmaceuticals to produce and distribute a formulation of 17-hydroxyprogesterone caproate under the trade name Makena™. With the availability of an FDA-approved formulation, an official at McGuff notified us that they would no longer be able to prepare medications for our trial. We considered the option of resuming the trial using Makena, but this would have resulted in a "blended" trial with medications obtained from 2 different sources. Although we had independent testing suggesting that the McGuff preparation was pure and stable, we felt that a blended trial would always be plagued by questions about the validity of combining subjects whose medications were obtained from two distinctly different sources. Thus we elected to terminate the present study and report the results. This decision was reviewed and approved by the DSMB.

Table 1 summarizes the baseline characteristics of those in each group. Table 2 summarizes the primary outcome and secondary outcomes related to pregnancy prolongation. Table 3 summarizes neonatal outcomes. Table 4 shows other outcomes of interest. Table 5 lists the participating sites. Additional File 2 presents the de-identified individual participant raw data.

**Table 1:** Baseline characteristics of study subjects

	17-Hydroxyprogesterone Caproate	Placebo
	**(N = 4)**	**(N = 8)**

Maternal Age (years)	28 ± 3	33 ± 6

Gestational Age at PROM (weeks)	25 ± 7	26 ± 4

Gestational Age at Randomization (weeks)	28 ± 3	27 ± 3

23.0-25.9 wks	1 (25%)	4 (50%)

26.0-28.9 wks	1 (25%)	1 (12.5%)

29.0-31.9 wks	2 (50%)	3 (37.5%)

Interval from PROM to Randomization (days)	3 (1-79)	1.5 (0-29)

Prepregnancy Weight (pounds)	157 ± 35	162 ± 37

Body Mass Index (kg/M^2^)	24 ± 7	27 ± 6

Body Mass Index > 30 kg/M^2^	1 (25%)	3 (37.5%)

Nulliparous	3 (75%)	5 (62.5%)

History of Prior Preterm Birth	0	0

Progestins Used Prior to 15 Weeks	1 (25%)	2 (25%)

Conception		

Spontaneous	3 (75%)	7 (87.5%)

*In Vitro *Fertilization	1 (25%)	0

Intrauterine Insemination	0	1 (12.5%)

Married/Living with Partner	3 (75%)	7 (87.5%)

College Education or More	1 (25%)	5 (62.5%)

Ethnicity		

Caucasian	3 (75%)	6 (75%)

African-American	1 (25%)	1 (12.5%)

Native American	0	1 (12.5%)

Reported Antepartum Substance Use		

Alcohol, Rare	0	1 (12.5%)

Marijuana	1 (25%)	1 (12.5%)

Tobacco	0	2 (25%)

Other	0	0

Results expressed as Mean ± SD, Median (range), or N (%).

**Table 2:** Primary outcome and gestational age outcomes

	17-Hydroxyprogesterone Caproate	Placebo
	**(N = 4)**	**(N = 8)**

Primary Outcome		

Delivery at 34.0 weeks or more	0	0

Delivery at 32.0-33.9 weeks with documented fetal lung maturity	0	0

Gestational Age at Delivery (weeks)	30 ± 4	28 ± 3

Delivery before 32 weeks	2 (50%)	7 (87.5%)

Delivery before 34 weeks	4 (100%)	8 (100%)

Pulmonary Maturity Testing		

Immature	1 (25%)	2 (25%)

Not Tested	3 (75%)	6 (75%)

Latency, Randomization to Delivery (weeks)	1.6 ± 1.0	1.3 ± 1.6

Less than 1 week	1 (25%)	5 (62.5%)

1.0 to 1.9 weeks	1 (25%)	1 (12.5%)

2.0 weeks or more	2 (50%)	2 (25%)

Reason for Delivery Before 34 weeks		

Spontaneous	0	4 (25%)

Chorioamnionitis	1 (25%)	2 (25%)

Fetal Indications	3 (75%)	2 (25%)

Results expressed as Mean ± SD or N (%).

Fetal indications for delivery included abnormal fetal heart tracings (4 cases) and fetal growth restriction with abnormal umbilical artery Doppler findings (1 case, placebo group).

**Table 3:** Neonatal Morbidity and Mortality

	17-Hydroxyprogesterone Caproate	Placebo
	**(N = 4)**	**(N = 8)**

Composite Neonatal Morbidity	3 (75%)	7 (87.5%)

Perinatal Death		

Stillbirth/Miscarriage	0	0

Neonatal Death	1 (25%)	1 (12.5%)

Respiratory Distress Syndrome	3 (75%)	7 (87.5%)

Sepsis, Culture-Proven	0	0

Pneumonia	0	0

Intraventricular hemorrhage, Grade 3 or 4	2 (50%)	0

Periventricular Leukomalacia	0	0

Necrotizing Enterocolitis	1 (25%)	0

Data expressed as N (%).

Composite Neonatal Morbidity = any one or more of the listed outcomes

**Table 4:** Selected other outcomes

	17-Hydroxyprogesterone Caproate	Placebo
	**(N = 4)**	**(N = 8)**

Maternal Management & Complications	3 (75%)	7 (87.5%)

Tocolysis in first 48 h	3 (75%)	5 (62.5%)

Antenatal Corticosteroids	4 (100%) *	8 (100%)

Cesarean Delivery	3 (75%)	5 (62.5%)

Preeclampsia or Gestational Hypertension	0	1 (12.5%)

Gestational Diabetes	0	2 (25%)

Chorioamnionitis	1 (25%)	1 (12.5%)

Sepsis	0	0

Neonatal Outcomes		

Birthweight, gms	1328 ± 547	1288 ± 525

Total Hospital Stay, Days	42 ± 23	57 ± 48

NICU Stay, Days	42 ± 23	56 ± 48

Newborns with Congenital Anomaly**	1 (25%)	3 (37.5%)

Adverse Events Not Tabulated Elsewhere***	0	1 (12.5%)

Data expressed as Mean ± SD or N (%)

NICU = Neonatal Intensive Care Unit

* One patient had received antenatal corticosteroids before PROM.

** One newborn in 17P group had ventriculoseptal defect and patent foramen ovale. Newborns with anomalies in the placebo group were 1 with bilateral inguinal hernias and hypospadias, 1 with unilateral inguinal hernia, and 1 with umbilical hernia.

*** One newborn had congenital lobar emphysema

**Table 5:** The obstetrix collaborative research network

Role	Personnel	Institutions
Investigative Sites	Rodney K Edwards MD, MS	Obstetrix Medical Group, Phoenix Perinatal Associates
	
	Melissa Ingersoll RN, CRC	Banner Good Samaritan Medical Center, Phoenix, AZ
	
	Ana Braescu, RN, MS	Banner Desert Samaritan Medical Center, Phoenix, AZ
		
		Banner Sun Health Research Institute (IRB)

Investigative Site	C Andrew Combs MD, PhD	Obstetrix Medical Group, San Jose
	
	Kimberly Mallory RN	Good Samaritan Hospital, San Jose, CA
	
	Stacey Maguire RN	Good Samaritan Hospital IRB

Investigative Sites	Richard Porreco MD	Obstetrix Medical Group of Colorado
	
	Kent Heyborne MD	Presbyterian Saint Luke's Hospital, Denver, CO
	
	Julie Rael RN	Swedish Medical Center, Englewood, CO
	
	Jeri Lech RN	Presbyterian Saint Luke's Hospital IRB
		
		HCA-HealthONE IRB

Investigative Site	David Luthy MD	Obstetrix Medical Group of Washington
	
	Tina Lopez RN	Swedish Medical Center, Seattle, WA
	
	Dawn Artis RN	Swedish Medical Center IRB

Investigative Site	George Lu MD	Obstetrix Medical Group of Kansas City
	
	Janice Etzenhouser RN	Saint Luke's Hospital of Kansas City, Kansas City, MO
		
		Saint Luke's Hospital of Kansas City IRB

Investigative Site	Wilson Huang MD	Center for Maternal Fetal Medicine
	
	Judy Hancock MSN	Sunrise Hospital, Las Vegas, NV
		
		Sunrise Health IRB

Investigative Site	Asad Sheikh MD	Spectrum Health Maternal Fetal Medicine
	
	Lori Oosterman BSN, RN	Spectrum Health Hospital, Grand Rapids, MI
	
	Alison Dutkiewicz RN	Spectrum Health Hospital IRB

Investigative Site	Michael Nageotte MD	Obstetrix Medical Group, Southern California
	
	Christine Preslicka BSN, RN	Long Beach Memorial Medical Center, Long Beach, CA
		
		MHS Research Administration (IRB)

Investigative Site	Hugh Miller MD	Obstetrix Medical Group of Arizona
	
	Diane Mercer RN, CCRC	Tucson Medical Center, Tucson, AZ
		
		Tucson Medical Center IRB

Investigative Site	David Lewis MD	University of Cincinnati School of Medicine
	
	Christine DeArmond RN	University Hospital, Cincinnati, OH
		
		University of Cincinnati IRB

Data & Safety Monitoring Board	Reese Clark MD	Pediatrix Medical Group CREQ, Piedmont, SC
	
	Jay D Iams MD	Ohio State University, Columbus, OH
	
	Brian M Mercer MD	MetroHealth Medical Center, Cleveland, OH
	
	Barbara Marusiak, RN, MSc	Pediatrix Medical Group, Phoenix, AZ

Biostatistics	Anita Das, PhD	Axistat, Inc., San Francisco, CA

Trial Administration	Kimberly Maurel RN, MS	Obstetrix Medical Group CREQ, Fountain Valley, CA
	
	Kimberly Mallory RN	Obstetrix Medical Group CREQ, Campbell, CA
	
	Diana Abril RN	Obstetrix Medical Group CREQ, Gilbert, AZ
	
	Thomas J Garite MD	Obstetrix Medical Group CREQ, Steamboat Springs, CO
	
	C Andrew Combs MD, PhD	Obstetrix Medical Group CREQ, Campbell, CA

CREQ = Center for Research, Education & Quality

IRB = Institutional Review Board

Description of Additional Data Files

There was one neonatal death in each group. In the 17P group, the mother experienced PROM at 23.7 weeks of gestation, was randomized at 24.0 weeks, was diagnosed with chorioamnionitis 2 days later, and underwent induction of labor. She delivered at 610 g male infant who ultimately developed necrotizing enterocolitis and died at 24 days of life. In the placebo group, the mother had PROM at 19.9 weeks, was randomized at 24.0 weeks and started spontaneous labor 8 days later. The 1000 g male infant had severe respiratory distress and died shortly after birth.

## Discussion

Because the trial was prematurely terminated, it is obviously underpowered to make conclusions as to the efficacy or safety of 17P in women with PROM.

Premature termination of a clinical trial raises serious ethical dilemmas. As noted in an editorial in The Lancet [24], "There is ... a moral responsibility to ensure that the commitments shown by physicians to their patients in the research setting and the commitments made by patients to advance clinical knowledge are not subject to the vagaries of commercial restructuring." Although vagaries in the manufacturing and approval of 17P were the cause of our premature trial termination, we were faced with the practical reality that these circumstances made it impossible for us to complete the trial using the formulation with which we started.

Lievre et al. [25] stated that enrollment of a patient into a research trial is "a moral contract by which the study (the investigators, the scientists, and the sponsor) agrees to use all possible means to make the patient's participation useful to the community." Certainly, had we chosen not to report the results from the 12 subjects who enrolled, their participation would not have been useful. Such a choice would not be ethically tenable. We have every confidence that those subjects who were assigned to receive 17P actually received pure, sterile 17P and that those assigned to placebo actually received a pure, sterile preparation that was not visibly distinguishable from 17P. That is, the observations here are truly valid, randomized, double-blinded, and placebo-controlled. Though we cannot make much of the results in isolation, it is our expectation that these 12 subjects ought to be included in future metaanalyses and systematic reviews concerning the use of 17P after PROM.

We have started a virtually identical study using the FDA-approved formulation of 17P.

## Conclusion

Premature termination of the study left us with a sample too small to reach conclusions about efficacy of 17P after PROM. Nonetheless, the results are valid and ought to be included in any future metaanalyses or systematic reviews of this topic.

## Abbreviations

17P: 17-hydroxyprogesterone caproate; DSMB: Data Safety and Monitoring Board; FDA: United States Food and Drug Administration; IRB: Institutional Review Board; NICU: Neonatal Intensive Care Unit; PROM: Preterm Rupture of Membranes; SD: Standard Deviation

## Competing interests

The Obstetrix Collaborative Research Network has received a donation of Makena™ and placebo from KV Pharmaceuticals for its new study of 17P after PROM, mentioned in the last paragraph of the discussion.

The authors declare no other competing interests.

## Authors' contributions

CAC drafted the protocol, manuscript, and FDA IND submission and participated in enrollment and data analysis. TJG made critical contributions to trial design, protocol, & manuscript, and provided administrative oversight. KM coordinated FDA IND submission, participated in drafting of informed consent documents, supervised site initiation visits and training of personnel, provided oversight of data monitoring and data cleanup, coordinated DSMB meetings and documents. KM made critical contributions to protocol logistics, FDA IND submission, & IRB documents and performed data quality monitoring. RKE, GL, and RP made critical contributions to trial design meetings, protocol finalization, and manuscript, participated in enrollment and contribution of data. AD provided biostatistical consultation during trial design and data analysis, and made critical contributions to manuscript preparation. All authors read and approved the final manuscript.

## Availability of supporting data

Please refer to the section "Description of additional data files" on the last page

## Supplementary Material

Additional file 1 **Protocol, version 1.6, 17P for PROM; 17-alpha-hydroxyprogesterone caproate (17P) for prolongation of pregnancy in women with preterm rupture of the membranes (PROM), double-blind randomized clinical trial; Final version of protocol, approved by IRB at each site**. Click here for file

Additional file 2 **Data, 17P for PROM; Obstetrix Medical Group data, 17-hydroxyprogesterone caproate for preterm rupture of membranes; Results table, de-identified raw data for each participant**. Click here for file
