# Supplementary material for: 17-Hydroxyprogesterone caproate to prolong pregnancy after preterm rupture of the membranes: early termination of a double-blind, randomized clinical trial
Source: BMC Res Notes. 2011 Dec 29;4:568. doi: 10.1186/1756-0500-4-568 (PMC3260323; doi:10.1186/1756-0500-4-568)
Supplement: Additional file 1 — Protocol, version 1.6, 17P for PROM; 17-alpha-hydroxyprogesterone caproate (17P) for prolongation of pregnancy in women with preterm rupture of the membranes (PROM), double-blind randomized clinical trial; Final version of protocol, approved by IRB at each site. [file 1756-0500-4-568-S1.PDF]

17-alpha-Hydroxyprogesterone Caproate (17P) for Prolongation of Pregnancy In Women with Preterm Rupture of the Membranes (PROM), Double-Blind Randomized Clinical Trial

C. Andrew Combs, MD, PhD, Principal Investigator

Thomas Garite, MD, Principal Investigator

Anita Das, PhD, Statistician

Kimberly Maurel, RN, MSN, Clinical Trial Manager

Obstetrix Medical Group

Center for Research & Education

Version 1.6

15 April 2010

IND # 107785

Table of Contents

1. Specific Hypotheses 3

2. Introduction 3

 2.1 Frequency & Impact of PROM 3

 2.2 Risks of Continuing the Pregnancy After PROM versus Risks of Premature Delivery 3

 2.3 Management Options While Attempting Expectant Management 5

 2.4 Progesterone for Prevention of Prematurity with Risk Factors other than PROM 7

 2.5 Rationale for Studying Progesterone in Women with PROM 9

3. Methods

 3.1 Study Design 11

 3.2 Inclusion Criteria, Outline 11

 3.3 Inclusion Criteria, Definitions & Details 13

 3.4 Screening & Enrollment Procedures 21

 3.5 Group Assignment, Study Medications 21

 3.6 Management Common to All Patients, Regardless of Group Assignment 22

 3.7 Study Outcomes 27

 3.8 Data Collection 28

 3.9 Data Analysis 28

 3.10 Data Safety & Monitoring Board (DSMB) 31

 3.11 Ethical, Legal, Administrative Aspects 34

 3.12 Compliance with Regulations & Conventions 40

4. References 42

1. Specific Hypotheses

When given to women with preterm rupture of the membranes (PROM), a weekly dose of 17-alpha-hydroxyprogesterone caproate (17P) will:

a. Increase the probability of continuing the pregnancy until a favorable gestational age

b. Increase the interval between randomization & delivery

c. Decrease neonatal morbidity

2. Introduction

2.1 Frequency & Impact of PROM

Preterm birth complicates over 12% of pregnancies in the USA (Martin et al., 2007). Prematurity is the leading cause of neonatal morbidity & mortality in nonanomalous newborns (Armstrong et al., 2005), accounting for an estimated 70% of neonatal mortality after correction for anomalies. Preterm rupture of the membranes (PROM) is the leading identifiable cause of prematurity (Mercer et al., 2000). PROM accounts for about one-third of all preterm deliveries and 18-20% of perinatal deaths in the USA (Caughey et al., 2007).

2.2 Risks of Continuing the Pregnancy after PROM versus Risks of Premature Delivery

When PROM occurs at very early gestational ages, the clinician must make a decision whether to attempt to prolong the pregnancy or whether to recommend prompt delivery. Both approaches carry substantial risk. Continuation of the pregnancy carries risks to the mother, notably a 15-30% risk of intrauterine infection and a 2-13% risk of postpartum infection (Garite and Freeman, 1985; Simpson and Harbert, 1985; Beydoun and Yasin 1986; Mercer, 1992; Caughey et al., 2008). Continuation also poses risks to the offspring, including the same risk of intrauterine

infection, a 4-12% risk of placental abruption (Gonen et al., 1989; Ananth et al., 1996), and a 5-10% risk of umbilical cord accident (cord compression leading to severe fetal heart deceleration, prolapse of the cord through the cervix, or prolapse through the vaginal introitus) (Combs et al., 2004). Any of these complications can be lethal to the offspring. However, intentional delivery to minimize these risks places the offspring at risk for a host of complications of prematurity, including respiratory distress (10-40%), intracerebral hemorrhage (up to 5%), necrotizing enterocolitis (2-6%), and others (Combs et al., 2004; Caughey et al., 2008). These complications can also be lethal or can cause permanent disability.

The strategy of continuing the pregnancy is commonly called "expectant management." Expectant management is a "balancing act" in which risks of premature delivery are compared to risks of continuing the pregnancy. If the gestational age is less than 34 weeks, if there is no evidence of infection, and if the fetal heart rate monitoring is reassuring, most clinicians conclude that the risks of premature delivery are greater than the risks of continuing the pregnancy and will therefore attempt to prolong the pregnancy.

A potential added benefit of expectant management is that this strategy allows for the occasional possibility that the membranes might reseal. Resealing occurs in about 3% of cases of PROM and is generally associated with favorable outcomes (Johnson et al., 1990).

During expectant management, gestational age steadily increases, and the balance naturally shifts toward favoring delivery. Once the gestational age reaches 34 weeks, the risk of lethal or permanent sequelae of prematurity or minimal, so most clinicians agree that delivery is

warranted (Caughey, 2008). In addition, because RDS accounts most of the mortality & major morbidity of premature birth, many clinicians will attempt to assess fetal lung maturity via amniotic fluid analysis at 32-34 weeks of gestation in patients with PROM and will recommend delivery if lung maturity is documented (Caughey, 2008).

Despite an attempt at expectant management, the majority of patients with PROM will be delivered within the first week or so (Bengtson et al., 1989; Dale et al., 1989). The common reasons for this include the onset of spontaneous preterm labor, development of intraamniotic infection, placental abruption, and umbilical cord accident (Combs et al., 2004). The "latency period" (the time from PROM until delivery) roughly follows a "half-life function" with a half-life of about one week (Amon et al., 1988; McGregor et al., 1991; Grable et al., 1996; Mercer et al., 1997). That is, about half the patients with PROM will remain undelivered for the first week, half of those remaining will remain undelivered for another week, half of those for yet another week, etc.

2.3 Management Options while Attempting Expectant Management

Because of the high risks of preterm delivery, of infection, of placental abruption, and of cord accidents, any of which can occur with little or no warning, patients with PROM are generally hospitalized until delivery (ACOG, 2007; Caughey et al., 2008).

Before attempting expectant management, it is imperative to rule-out the possibility that intraamniotic infection (IAI) might already be present. It is generally agreed that the presence of IAI is a contraindication to expectant management. Clinical signs of IAI such as fever, uterine

tenderness, and foul-smelling vaginal discharge have notoriously poor sensitivity and specificity. For this reason, many clinicians perform amniocentesis to evaluate whether IAI is present (Caughey, 2008). Amniotic fluid can be examined for indirect markers of IAI (elevated white blood count (WBC), low glucose) as well as direct markers (gram stain showing organisms, positive culture). Many of the study sites for the present study routinely recommend amniocentesis to rule-out IAI in PROM patients. However, amniocentesis is possible in only about half of patients after PROM, largely because of the lack of a sufficient residual amniotic fluid in many patients (Porreco, 2008).

When attempting expectant management, several additional management options may be considered. These include prophylactic use of antibiotics to reduce the risk of intraamniotic infection, use of antenatal corticosteroids to accelerate fetal lung maturation, and use of tocolytic agents to suppress uterine contractions. In the mid-1980's it was unclear whether any of these produced benefit, leading Garite (1985) to refer to the management of PROM as "the enigma of the obstetrician." Since then, it has been shown that prophylactic antibiotics can reduce the risks of both maternal and neonatal infection, thereby resulting in a prolonged latency period and decreased neonatal morbidity (Mercer et al., 1997; Kenyon et al., 2001). Therefore, antibiotic prophylaxis has become standard in the expectant management of PROM (ACOG, 2007; Caughey et al., 2008). A multivariate analysis of prospective observational studies concluded that antenatal corticosteroids are beneficial after PROM (Wright et al., 1995). Considering that analysis and other data, a National Institutes of Health Consensus Panel (NIH, 2001) and the American College of Obstetricians & Gynecologists (ACOG, 2002; ACOG, 2007) recommend administration of corticosteroids to mothers with PROM prior to 32 weeks of gestation.

Tocolytic agents appear to be largely ineffective at either prolonging pregnancy or reducing neonatal morbidity after PROM (Combs et al., 2004), though many clinicians will use them for up to 48 hours to attempt to optimize the benefit of corticosteroids.

No interventions other than antibiotic prophylaxis or corticosteroids have been shown to prolong latency or reduce neonatal morbidity after PROM.

2.4 Progesterone for Prevention of Prematurity with Risk Factors other than PROM

Recent evidence suggests that prophylactic administration of progesterone medications may reduce the risk of preterm delivery in women with certain risk factors, notably those with a history of a prior preterm delivery and those with a shortened cervix discovered by ultrasound examination. A metaanalysis of trials performed in the 1960's through the 1980's concluded that progesterones as a group were not effective in preventing preterm birth (Goldstein, 1989). However, a subanalysis focusing on 17-alpha-hydroxyprogesterone caproate (17P) found that this medication caused a reduction in prematurity, especially in those with a history of prior preterm birth (Keirse, 1990).

In 2003, the NIH Maternal-Fetal Medicine Units (MFMU) Network reported on the largest trial to date of 17P in women with a history of prior preterm birth (Meis et al., 2003). They found that 17P (250 mg IM weekly, started at 18-19 weeks of gestation on average) reduced the risk of recurrent preterm birth by about 40% (rate of preterm delivery 54% with 17P versus 36% with placebo.)

Also in 2003, da Fonseca et al. reported that on the use of natural progesterone (100 mg vaginal suppositories daily, started at an average of 25-27 wks of gestation) given to women at high risk of preterm birth (94% of the participants had a history of prior preterm delivery.) They found that progesterone reduced the risk of recurrent preterm birth by more than 50% (14% with progesterone vs. 29% with placebo)

In November, 2003, the ACOG Committee on Obstetric Practice issued a Committee Opinion reviewing these trials and concluding that progesterone "reduces preterm birth in a select very high-risk group," evidently referring to those with a history of prior preterm birth (ACOG 2003). The Opinion noted that the optimum formulation was not known (17P IM vs. progesterone suppositories.) Since then, another trial showed that a novel progesterone suppository with a bioadhesive gel was not effective at reducing recurrence of preterm birth (O'Brien et al., 2007). The Opinion noted that additional research was needed to determine whether progesterone was effective in reducing preterm birth in women with risk factors other than a prior preterm birth, including multiple gestations, women with a shortened cervix, those with a positive fetal fibronectin examination.

Since 2003, there has been an explosion of interest in 17P and other progesterones in women at risk for preterm delivery. Two studies found that vaginal progesterone reduced the risk of preterm delivery in women with a shortened cervix found on ultrasound examination (Defranco et al., 2007; Fonseca et al., 2007). A recent clinical trial found that 17P did not reduce the risk of preterm delivery in twin pregnancy (Rouse et al., 2007). Worldwide, there are several other ongoing trials of 17P in twin pregnancy (including our own trial which has recently completed

enrollment). In triplet pregnancy, 17P was not beneficial in reducing prematurity (Caritis et al., 2009). Progesterone therapy has been attempted after arrest or preterm labor, with variable results (Tita & Rouse, 2009).

2.5 Rationale for Studying Progesterone in Women with PROM

Clearly, women with PROM are at very high risk of preterm delivery, so there is a pressing need to study whether 17P is effective after PROM. To our knowledge, there are no ongoing or completed trials of 17P in women with PROM. The NIH website clinicaltrials.gov lists 17 ongoing studies of progesterone in various settings (prior preterm birth, short cervix, twins, triplets, threatened preterm labor), but none concerning PROM.

Progesterone might be beneficial after PROM both because it tends to promote uterine quiescence by suppressing the formation of myometrial gap junctions and because it has anti-inflammatory properties, suppressing the production of inflammatory cytokines and thereby inhibiting cervical ripening (Romero, 2007). Inflammation is a major pathway leading to preterm labor, cervical dilation & preterm delivery.

Regarding safety during pregnancy, progesterone has been extensively studied & found to be safe. A Cochrane review found no evidence that progesterone had any adverse effects including stillbirth, neonatal death, low birth weight, fetal genital abnormalities, other teratogenic effects, or admission to special care units (Oates-Whitehead, 2004). A California Environmental Protection Agency review documented some concerns regarding potential for potential genital abnormalities with progesterone given in the 1st trimester (Golub et al., 2004), but these concerns

are irrelevant to the present study in which 17P will only be given after 23 weeks of gestation. In the large NIH MFMU Network trial, the only adverse reactions noted were local irritation at the injection site (Meis et al., 2003).

In order to be useful in the setting of PROM, it is essential that 17P must be effective when started and continued relatively late in the pregnancy. Two retrospective analyses suggested that the efficacy of prophylactic 17P is comparable whether the medication is started early (prior to 21 weeks of gestation) or late (after 21-27 weeks; Gonzalez-Quintero et al., 2007; How et al., 2007.) Indeed, in the da Fonseca trial (2003), vaginal progesterone was not started until 24 weeks' and mean gestational age at enrollment was 25-27 weeks. Another retrospective analysis showed that cessation of 17P prior to 32 weeks of gestation resulted in higher preterm delivery rates than continuation until 36 weeks, suggesting that the medication remains effective relatively late in the pregnancy (Rebarber et al., 2007.) Thus, 17P seems like an ideal candidate for prolongation of pregnancy after PROM.

3. Methods

3.1 Study Design

This is a double-blinded, placebo-controlled, multicenter, randomized clinical trial of 17P versus placebo. The primary outcome measure will be the percentage of each group reaching either a gestational age of 34w0d or documentation of fetal lung maturity at 32w0d to 33w6d. Secondary outcomes will include the latency period for each group and the percentage of newborns in each group who have major neonatal morbidity or death.

3.2 Inclusion Criteria, Outline

Definitions of terms and details regarding these criteria are elaborated in the next section, starting on page 13.

1. Participant is 18 years old or older
2. Gestational age is between 23w0d and 31w6d, inclusive, at time of enrollment
3. Singleton pregnancy
4. PROM defined as either (a) or (b) or (c) below
   (a) Documentation of vaginal leakage of indigo carmine dye instilled via amniocentesis
   (b) Positive Amnisure® test
   (c) Two or more of (i) through (iv):
      i. Nitrazine test with pH of 7 or more
      ii. Positive fern test
      iii. Gross pooling of clear fluid in vaginal posterior fornix
      iv. Ultrasound exam showing oligohydramnios
5. No contraindication to expectant management, including

a. Suspected intraamniotic infection

b. Active preterm labor

c. Nonreassuring fetal heart rate tracing

d. Intrauterine fetal death

e. Preeclampsia

f. Active bleeding from uterus

g. Fetal lung maturity documented on amniotic fluid specimen prior to enrollment

6. No fetal condition likely to cause serious neonatal morbidity independent of gestational age, including:

a. Malformation likely to require surgery

b. Malformation involving vital organs

c. Fetal viral infection

d. Hydrops fetalis

7. No history of allergy to 17P or the oil vehicle

8. No medical condition that might adversely interact with 17P

a. asthma requiring medications

b. renal insufficiency (serum creatinine 1.2 mg/dl or more)

c. seizure disorder

d. ischemic heart disease

e. active cholecystitis

f. impaired liver function

g. history of venous thromboembolism

h. history of breast cancer

i. history of depression requiring hospitalization

9. No medical condition treated with systemic steroid medications

10. No cervical cerclage present at the time of PROM

11. Informed consent obtained & signed

3.3 Inclusion Criteria, Definitions & Details

1. Participant is 18 years old or older. Age is determined at the time of enrollment. The purpose of this criterion is to ensure that the research participant is an adult who can give her own informed consent. Even though many states consider pregnant women under 18 years old to be "emancipated minors" this study requires the mother to be 18 or older to ensure uniformity across study sites.

2. Gestational age is between 23w0d and 31w6d, inclusive, at time of enrollment.

Gestational age is expressed as N weeks plus n days, abbreviated Nwnd (example 31w6d). Gestational age is based on a combination of last menstrual period (LMP) and earliest reliable ultrasound examination dating. If the LMP is unknown or uncertain, ultrasound dating will be used. With a known LMP, LMP dating will be used if the LMP dating is in agreement with the ultrasound dating within these tolerances:

a. earliest reliable ultrasound exam before 10w0d within 4 days of LMP

b. earliest reliable ultrasound exam at 10w0d to 17w6d within 7 days of LMP

c. earliest reliable ultrasound exam at 18w0d to 25w6d within 10 days of LMP

d. earliest reliable ultrasound exam at 26w0d to 31w6d within 14 days of LMP

If the LMP and earliest ultrasound are not in agreement, then the dating will be based on earliest reliable ultrasound. A patient with PROM at 22w6d or less is not eligible, but may become eligible at 23w0d if she still meets all other criteria. A patient at 31w6d is eligible. A patient at 32w0d is not eligible.

3. Singleton pregnancy. A single living fetus is present. If there is a history of twin pregnancy in the 1st trimester with demise of one twin before 12w0d, this will be considered a singleton pregnancy. Demise of a twin at 12w0d or beyond or demise of triplets or higher order multiple gestations at any gestational age will make a patient ineligible for randomization. Multifetal pregnancy reduction (iatrogenic fetal demise) will be considered equivalent to spontaneous demise for purposes of this inclusion criterion. Thus, a patient who has undergone reduction of twins to singleton before 12w0d will meet this criterion, but a patient who has undergone reduction of triplets to singleton will not.

4. Definition of PROM. PROM is defined as either (a) or (b) or (c) below

(a) Documentation of vaginal leakage of indigo carmine dye instilled via amniocentesis. If the physician performs amniocentesis for assessment of possible intraamniotic infection and/or for assessment of fetal lung maturity, then 1 ml of sterile indigo carmine dye diluted with 9 ml sterile saline will be injected into the amniotic sac at the end of the procedure. If the dye is found on a vaginal tampon or perineal pad within 6 hrs, this establishes the diagnosis of PROM. If amniocentesis is not performed, then the other tests may be used.

(b) Positive Amnisure® test. The Amnisure® test will be used at sites where it is clinically available. The test will be performed by an RN or by a laboratory technologist with documented current competency per standard practice at each site. If Amnisure® test is not in use or is unavailable at the site, then the other tests may be used.

(c) Two or more of (i) through (iv):

i. Nitrazine test with pH of 7 or more. Nitrazine testing will be performed with a sterile cotton swab moistened in the posterior vaginal fornix during speculum examination. The test will be interpreted by a physician with reference to the pH scale on the nitrazine paper container.

ii. Positive fern test. The swab used for nitrazine testing will be streaked on a glass slide. The slide will be air-dried and examined for a typical “fern” pattern (“arborization”) by a physician or laboratory technologist with current competency.

iii. Gross pooling of clear watery fluid in vaginal posterior fornix. This assessment will be made by a physician during speculum vaginal examination.

iv. Ultrasound exam showing oligohydramnios. Oligohydramnios is defined as a 4-quadrant amniotic fluid index of 5 cm or less, or by the absence of any single vertical amniotic fluid pocket of 2 cm or more.

<u>5. No contraindication to expectant management</u>, Contraindications include, but are not limited to the following:

a. **Suspected intraamniotic infection** (IAI). Clinical suspicion of IAI is based on a maternal temperature of 100^0F or more PLUS one or more of the following:

(i) white blood count (WBC) of 15,000 per mm^3 or more

(ii) uterine tenderness

(iii) malodorous or purulent vaginal fluid discharge

(iv) maternal tachycardia, baseline heart rate 101 or more

(v) fetal tachycardia, baseline heart rate 161 or more

** If present, the patient will not be eligible for randomization. Many of the study sites routinely recommend amniocentesis if possible in all patients with PROM to evaluate for possible IAI. However, amniocentesis is not possible in about half of cases, owing to lack of sufficient amniotic fluid volume. If amniocentesis if performed, the suspicion of IAI is based on:

(i) gram stain showing bacteria or yeast

(ii) positive culture

(iii) WBC 100 per mm^3 or more

(iv) glucose less than 20 mg/dL

** If any of these 4 is present, the patient will not be eligible for randomization.

** Note that the suspicion of IAI is used to determine whether a patient should be entered into the study. Investigators should not randomize patients who have not been fully evaluated for possible IAI. That is, if amniocentesis is planned, or if the immediate results are pending, the patient should not be randomized until the results are available. This does not preclude study personnel from discussing the study & obtaining consent while results are still pending. Nor does it preclude randomizing a

patient in whom the WBC, glucose, and gram stain do not show infection but in whom the culture results are pending. Such patients will normally be managed expectantly for several days pending the culture results and may be randomized assuming they meet all other inclusion criteria. Once randomized, all patients will be analyzed by the “intent to treat” approach and will not be excluded later. Thus, if IAI is diagnosed after randomization, the patient will still be evaluable.

b. **Active preterm labor**. If the cervix is 4 cm dilated or more, there is little hope of any reasonable period of expectant management and the patient should not be randomized. Digital examination of the cervix is generally discouraged after PROM, so the assessment of cervical dilation will usually be based on visual inspection via a speculum examination. Similarly, if there are persistent strong, regular uterine contractions (8 or more per hour, perceived by the patient), the patient should not be enrolled. If the uterus later becomes quiescent (with or without tocolysis, see below) and the cervix is less than 4 cm dilated, she may be randomized at that time.

c. **Nonreassuring fetal heart rate tracing.** This is a clinical diagnosis left to each site investigator. If the investigator feels that the fetal tracing requires delivery, then the patient is not eligible for study.

d. **Intrauterine fetal death**. If there is no living fetus, there is no reason to continue the pregnancy after PROM.

e. **Preeclampsia**. Although expectant management is sometimes attempted with preeclampsia at early gestational ages, the presence of preeclampsia with PROM suggests a higher than usual likelihood of imminent delivery. According to the diagnostic criteria of the American College of Obstetrics & Gynecology, preeclampsia is defined as hypertension plus abnormal proteinuria. Hypertension is defined as systolic blood pressure (BP) 140 mmHg or higher or a diastolic BP 90 mmHg or higher on two occasions at least 6 hrs apart. Abnormal proteinuria is defined by 300 mg total protein or more in a 24 hr collection (or 1+ protein or more on dipstick if a 24 hr urine is not available.)

f. **Active bleeding from the uterus**. If bleeding is such that the attending physician feels that prompt delivery is indicated, the patient should not be enrolled in the study. If there has been brisk bleeding after PROM but the bleeding has stopped, the patient may be enrolled & randomized if the attending physician feels that expectant management is reasonable.

g. **Fetal lung maturity** documented on amniotic fluid specimen prior to randomization. Such patients should not be enrolled because they have already achieved a "favorable endpoint" as defined in the first specific hypothesis of the study.

<u>6. No fetal condition likely to cause serious neonatal morbidity independent of gestational age.</u>

Such conditions would be expected to adversely impact neonatal outcome regardless of the

duration of the pregnancy and thus patients known to have such conditions should not be randomized. Such conditions may include, but are not necessarily limited to:

a. Fetal malformation likely to require surgery (examples: abdominal wall defect, neural tube defect, sacrococcygeal teratoma, structural cardiac defect)

b. Fetal malformation involving vital organs (examples: severe hydrocephalus, transposition of great arteries)

c. Fetal viral infection

d. Hydrops fetalis

7. <u>No allergy to 17P or the oil vehicle</u>. Patients with such allergies should not be enrolled into the study or given any study medications.

8. <u>No medical condition that might adversely interact with 17P</u>. Patients with such conditions should not be randomized or given any study medications. Such conditions may include, but are not necessarily limited to:

a. asthma requiring medications

b. renal insufficiency (serum creatinine 1.2 mg/dl or more)

c. seizure disorder (except isolated history childhood febrile seizure)

d. ischemic heart disease

e. active cholecystitis

f. impaired liver function

g. history of venous thromboembolism

h. history of breast cancer

i. history of depression requiring hospitalization

9. No medical condition treated with systemic steroid medications. Patients with conditions such as rheumatologic disorders, inflammatory bowel disease, or others treated with oral or parental steroids should not be enrolled. In the present study, all patients will be given a course of glucocorticoids to accelerate fetal lung maturity. We will exclude patients on chronic steroid therapy because there is no generally accepted protocol for substitution of medications while administering antenatal glucocorticoids. Such patients are expected to be uncommon.

10. No cervical cerclage in place at the time of PROM. Patients who rupture membranes with cerclage in situ are excluded from randomization for 2 reasons. First, they comprise an unusual group (those with incompetent cervix). Second, there is no clear consensus regarding whether the cerclage should be left in situ or removed after PROM. Indeed, this question is the participant of another randomized trial by our group.

11. Informed consent obtained. Before randomization, study personnel will explain the study to a potential participant, including risks, benefits, & alternatives and will have the participant sign an informed consent document approved by the site-specific Institutional Review Board (IRB.) An example consent document is attached.

3.4 Screening & Enrollment Procedures

Each site will maintain a screening log showing all patients admitted with PROM. The log will record a disposition for each patient, from the following checkbox choices:

1. not eligible for trial (one or more inclusion criteria not met, list criteria not met)
2. eligible but not approached for enrollment
3. approached but patient declined to consent
4. informed consent obtained but not randomized
5. randomized

With the permission of the patient's attending physician, eligible patients will be approached by study personnel, will receive an explanation of the study procedures, risks, benefits & alternatives, and will be asked to voluntarily consent to the study. If consent is obtained and appropriately signed, dated, and witnessed, an order will be recorded in the medical record directing the inpatient pharmacy to assign a randomization code to the patient and to dispense a weekly dose of study medication (either 17P or placebo.) The enrolled participant will be entered onto an enrollment log and capture the enrollment information.

3.5 Group Assignment, Study Medications

Each patient will be randomly assigned to one of two treatment arms, 17P or placebo.

a. Progesterone (17P) group. Patients in the 17P group will be given 17P, 250 mg IM weekly in 1 ml of oil vehicle.

b. Placebo group. Patients in the placebo group will be given a weekly IM injection of 1 ml of oil vehicle.

Patients, investigators, and study personnel will be blinded as to group assignment. A computer randomization program will be used to generate a randomization code book which will be kept at the pharmacy at each study site hospital. The pharmacy will use the code-book to determine which medication to dispense (17P or placebo) to the patient. The code will not be broken or revealed until all patients have completed the study & their data submitted, unless the data safety/monitoring committee concludes that breaking the code is necessary to evaluate an adverse event.

To ensure balanced group assignments at each study site, randomization will be stratified in 3-week gestational age strata (23w0d-25w6d, 26w0d-28w6d, 29w0d-31w6d).

The assigned medication will be administered by a registered nurse. The administration will be recorded in the inpatient medical record, for example, "Study medication, progesterone or placebo, 1 ml, IM injection in right ventral gluteus" or similar notation as required per hospital policy at each site. The research personnel will also document the medication administration on the participant's case report form..

3.6 Management Common to All Patients, Regardless of Group Assignment

a. Hospital admission until delivery. All enrolled patients will be admitted for inpatient management until delivery. An exception will be made if the membranes reseal, as discussed at the end of this section.

Antenatal corticosteroids. All patients will be given a single course of antenatal corticosteroids with an intention to accelerate fetal lung maturation. A "course" of corticosteroids is defined as either:

(a) two doses of betamethasone, 12 mg IM, given at 24 hr intervals OR

(b) four doses of dexamethasone, 6 mg IM, given at 12 hr intervals

Betamethasone is preferred. Dexamethasone may be used if betamethasone is unavailable or if the patient has already been started on dexamethasone. If the patient received a complete course of corticosteroids at any time after 22w0d and before enrollment, no further corticosteroids will be given. That is, she will not be given so-called "rescue steroids". If the patient received a partial course within the 2 days prior to enrollment, the remaining dose(s) of the same medication will be given. If the patient received a partial course more than 2 days prior to enrollment, a complete course will be given upon enrollment.

Prophylactic antibiotics. All patients will receive a course of antibiotic prophylaxis. Each study site has a slightly different standard regimen for prophylaxis after PROM, but all use broad-spectrum antibiotics for 5-7 days in regimens similar to that of Mercer et al. (1997). The prophylactic antibiotics used, dosage, and route of administration will be recorded in the case report form for each participant.

Antibiotics for Labor & Delivery. Antibiotic therapy is frequently used during labor or frequently used preoperatively for cesarean delivery in patients with PROM. The choice of antibiotic regimen for these indications is left to the patient's attending physician. The dosage

and route of peripartum antibiotics used, if any, will be recorded in the case report form for each participant.

Tocolytic therapy. Medications intended to suppress contractions may be used at the discretion of the attending physician during the first 48 hrs after administration of the first dose of corticosteroids. Thereafter, such medications will not be used. These medications include magnesium sulfate, terbutaline, nifedipine (and other calcium channel blockers), & indomethacin (and other cyclooxygenase inhibitors or nonsteroidal anti-inflammatory drugs such as ibuprofen, celicoxib, or sulindac.)

Amniocentesis to rule-out intraamniotic infection (IA). We encourage investigators to perform amniocentesis & to evaluate the preliminary ("stat") results before randomizing patients into this study. After randomization, amniocentesis may become clinically indicated to evaluate suspected IAI in some cases. If performed, fluid will be sent for gram stain, glucose, WBC, and culture, with interpretation as per the discussion above, and for fetal lung maturity studies if performed between 32w0d and 33w6d.

Fetal heart rate monitoring. At least one hour of fetal monitoring every day will be performed. Additional monitoring will be allowed per the discretion of the patient's attending physician.

Avoidance of Digital Examination of the Cervix. Because insertion of a gloved finger into the cervix might introduce vaginal bacteria into the uterus, thereby increasing the risk of intraamniotic infection, digital examination of the cervix is generally discouraged while

attempting expectant management of PROM. Most obstetricians have been trained to examine the cervix by visual inspection via speculum examination. This is the preferred method for patients in this study. The number & timing of digital examinations & speculum examinations performed before the onset of active labor will be tabulated in the case report form for each participant.

Vaginal amniotic fluid specimen for FLM at 32 wks, if possible. If the patient reaches a gestational age of 32w0d during expectant management & if she is still leaking amniotic fluid, an attempt will be made to obtain a sample of fluid leaked from the vagina for assessment of fetal lung maturity. A fluid sample collected by amniocentesis may be used in lieu of a vaginally collected sample if there is an indication for amniocentesis at 32w0d or more. Each study site will use its customary test for fetal lung maturity. Tests in use at our study sites include TDX-FLM2™, lecithin/sphingomyelin ratio, phosphatidylglycerol, and lamellar body counts. If fetal lung maturity is documented, delivery will be initiated. If it is not possible to obtain a fluid specimen or if the fluid does not indicate lung maturity, then expectant management will be continued until 34w0d of gestation & delivery initiated at that time. All study site investigators agree that continued expectant management is not reasonable after 34 weeks of gestation. Beyond 34 wks, the risks of premature delivery are small compared to the risks of attempted expectant management (IAI, cord accident, and abruption, any of which can be lethal.)

Route of Delivery. Vaginal delivery will ordinarily be attempted unless there is a standard obstetric indication for cesarean delivery, including malpresentation, abnormalities of the fetal

heart rate tracing, or a history of prior cesarean delivery. The route of delivery will be at the discretion of the attending physician in consultation with the patient.

Membrane resealing. To diagnose resealing, the following 3 criteria must all be present (a, b, and at least one part of c).

a. the patient reports cessation of fluid leakage for at least 72 hrs, and then no fluid leakage occurs after a 1-day trial of light ambulation

b. ultrasound exam shows normal amniotic fluid volume (four-quadrant fluid index of 8 cm or more)

c. at least ONE of the following confirmatory tests is documented:

i. Amnisure (TM) of cervicovaginal secretions is negative

ii. Fetal Fibronectin test of cervicovaginal secretions is negative

iii. Speculum examination shows no pooling of fluid and fern and nitrazine tests are both negative.

iv. Indigo carmine dye instilled in the amniotic sac via amniocentesis does not leak vaginally within 24 hrs

If membrane resealing is documented, the patient will be discharged from the hospital for self-care at home. The study medications (progesterone vs placebo) and prophylactic antibiotics will be discontinued upon discharge from the hospital. An attempt will be made to capture delivery & neonatal outcome data, even if delivery occurs at another hospital. For analysis under the "intent-to-treat" principle, the patient will be included with her originally-assigned group (progesterone or placebo).

3.7 Study Outcomes

1. Primary outcome: Pregnancy continues until 34w0d of gestation or documentation of lung maturity at 32w0d to 33w6d.

2. Secondary outcomes:

   a. duration of latency period (time from randomization to delivery)

   b. composite neonatal morbidity, defined as one or more of the following, using the definitions of Mercer et al. (1997):

      i. stillbirth

      ii. neonatal death

      iii. infant death before hospital discharge

      iv. respiratory distress syndrome (RDS)

      v. intracranial hemorrhage (ICH), grade 3 or 4

      vi. necrotizing enterocolitis (NEC), stage 2 or 3

      vii. documented neonatal sepsis within 72 hr of birth

      viii. periventricular leukomalacia (PVL): characteristic lesions in the subcortical white matter seen on cerebral imaging studies within 96 hrs of birth (not included in the definitions of Mercer et al. (1997))

3. Tertiary outcomes

   a. gestational age at delivery

   b. route of delivery

   c. neonatal length of stay

   d. birth weight

   e. Apgar scores

f. Amnionitis

g. Postpartum endometritis

3.8 Data Collection

A Case Report Form (CRF) will be used to capture all primary, secondary, and tertiary data. The original CRF will remain in a locked file at the study site and a copy will be faxed to the central data center for computer entry. The CRF will reflect events until the mother & newborn are both discharged from the hospital. If the infant remains hospitalized at 60 days after birth, the CRF will reflect all events in the first 60 days of life.

3.9 Data Analysis

Primary Outcome. The primary outcome measure is the proportion of women who continue the pregnancy to a favorable gestational age (34w0d or documented fetal lung maturity at 32w0d to 33w6d). The null and alternative hypotheses are as follows:

H0: $p_1 = p_2$ and H1: $p_1 \neq p_2$

where p1=the proportion of women who continue the pregnancy to a favorable gestational age in the progesterone group and p2=the proportion of women who continue the pregnancy to a favorable gestational age in the placebo group

To test the null hypothesis, a Cochran-Mantel-Haenszel test will be used, stratified by gestational age group at randomization. The final alpha level will be based on the O'Brien-Fleming spending function and depends on the number and timing of interim analyses. If, as planned, only one interim analysis occurs at 50% of participants randomized, the final alpha level will be

0.049. The primary outcome measure will be analyzed in the intent-to-treat population (i.e. all randomized participants).

Secondary Outcomes. The duration of latency will be determined as the time from randomization until delivery. A staggered entry Kaplan-Meier analysis (which adjusts for gestational age at randomization) will be completed with participants with missing outcome data censored on the date last known pregnant. Significant differences between the progesterone and placebo group will be determined using the log-rank test.

The percentage of participants with at least one of the neonatal morbidity measures (composite neonatal morbidity) will be tested for significant differences between the treatment groups using a Cochran-Mantel-Haenszel test stratified by gestational group at randomization. The percentage of participants with each of the component morbidities (e.g. RDS, NEC, IVH, etc.) will be similarly compared.

All secondary outcome measures will be tested for statistical significance using an alpha level of 0.05. Latency and composite neonatal morbidity will be analyzed in the intent-to-treat population. With the exception of stillbirth (analyzed in the intent-to-treat population), the component morbidities will be analyzed in all participants with a live birth.

Sample Size & Power Calculations. A total of 222 pregnant women (111 in each treatment group) will be randomized in the study. A total sample size of 105 participants is sufficient to detect an absolute increase in the proportion of women reaching a favorable gestational age of

20% (from 30% in the placebo group to 50% in the progesterone group). This sample size was calculated assuming a type I error (2-sided) of 5% and a power of 80% and adjusting for one interim look at 50% of participants randomized. Because PROM patients are hospitalized until delivery, loss-to-follow-up should be minimal, comprising only patients whose membranes reseal & who deliver elsewhere. The planned sample size was increased from 105 patients per group to 111 patients per group to compensate for a loss-to-follow-up rate of 5% or less.

The proposed sample size is also sufficient to detect a 4-day between-group difference in latency, assuming a standard deviation of 10 days with a power of approximately 80%.

The proposed sample size is also sufficient to detect a reduction in neonatal morbidity from 60% in the placebo group to 40% in the 17P group with a power of approximately 80%.

Interim Analysis. One interim analysis of efficacy will be conducted when 50% of participants have outcome data available (111 participants total). The group sequential method of Lan and DeMets will be used to characterize the rate at which the type I error is spent where the spending function is the O'Brien-Fleming boundary. The nominal alpha level at the interim analysis (50% of participants) is 0.0031 and at the final analysis (100% of participants) is 0.049. In addition, a futility analysis will be conducted whereby the probability of finding a significant result at the end of the study will be calculated based on the current trend in the data, the null and alternative hypotheses.

3.10 Data Safety & Monitoring Board (DSMB)

The DSMB will comprise physicians & nurses familiar with the study but not participating as investigators or employed at any of the study sites. The DSMB will meet on an ad hoc basis and as needed to perform its various functions. Most meetings will be by conference telephone call or by electronic mail conference. The DSMB will have the following functions and the study will follow the following data safety plan:

a. Review of all Adverse Event (AE) and Serious Adverse Event (SAE) reports. Such review will occur within 10 days of receipt of an AE report and within 24 hours of receipt of an SAE report. The DSMB will make an independent determination as to whether each SAE was likely related or unrelated to the study medications or procedures and will make a determination as to whether the group assignment code ought to be broken to further evaluate the event.

- The principal investigator or designee at each study site is responsible to report any SAE to the Obstetrix Center for Research & Education (CRE) and to the DSMB within 24 hrs of recognition of the event. The report will be made using a standardized form submitted by fax or by e-mail to the study coordinator. SAE's include these events:
  - (i) Anaphylactic reaction or other life-threatening reaction to injection of study medication (17P or placebo)
  - (ii) Injection site infection requiring incision & drainage or IV antibiotic therapy

- The principal investigator or designee at each study site is responsible to report any AE to the Obstetrix CRE and to the DSMB within 10 days. The report will be made

using a standardized form submitted by fax or by e-mail to the study coordinator. AE's include these events:

(i) Injection site infection treated with oral antibiotics

(ii) Pain or other symptom at injection site that causes the mother to discontinue further injections.

- Neonatal morbidity events attributed directly to preterm delivery and/or to perinatal infection are specifically excluded from the list of adverse events. Indeed, such events are common & expected in patients with PROM. They comprise an important set of endpoints in this trial. Their frequency will be analyzed with the tertiary hypothesis. Such events include, but are not necessarily limited to:

    (i) death of newborn born at gestational age 28w0d or less

    (ii) respiratory distress syndrome (RDS)

    (iii) intracranial hemorrhage (ICH)

    (iv) necrotizing enterocolitis (NEC)

    (v) positive newborn blood culture

    (vi) suspected neonatal sepsis

- Similarly, maternal morbidities known to be associated with PROM are specifically excluded from the list of adverse events. Such events include:

    (i) prolonged hospitalization

    (ii) intraamniotic infection

    (iii) placental abruption

b. Planned interim analysis. Upon receipt of 50% of the CRF's (111 participants total), the DSMB will conduct an interim analysis of the efficacy & safety results. The primary outcome will be compared between the treatment groups to determine if there is a significant difference. The frequency of SAE's and AE's in the two groups (17P vs. placebo) will also be compared. Based on the results of this analysis, the DSMB will make a determination whether the trial should continue. The trial will be discontinued if any one of the following is found:

(i) A significant between-group difference is found in the proportion of participants with the primary outcome measure. At the interim analysis (50% of participants), significance is defined as alpha = 0.0031.

(ii) It seems extremely unlikely that continuation of the trial will disclose a significant difference based on the conditional probability analysis.

(iii) There is a safety concern, such as significantly higher rate of AE's, SAE's, or neonatal morbidity in the 17P group.

If the trial is discontinued, all enrollment & randomization of new participants will be immediately halted. A final data analysis will be completed after the CRFs have been submitted from all randomized participants. If the trial is continued, the details of the interim analysis will not be made available to the site investigators, but a summary report will be issued to the site investigators stating that the DSMB concluded that continuation of the study was warranted.

3.11 Ethical, Legal and Administrative Aspects

a. Data Quality Assurance. Obstetrix research staff or their designee will conduct a site visit to verify the qualifications of each investigator, inspect the site facilities, and inform the investigator of responsibilities and the procedures for ensuring adequate and correct documentation.

Each investigator is required to prepare and maintain adequate and accurate case histories designed to record all observations and other data pertinent to the study for each study participant. All information recorded on the CRFs for this study, or on correction sheets (query forms or data clarification forms – DCFs) must be consistent with the participants' source documentation (i.e., medical records).

b. Case Report Forms and Source Documentation. All data obtained during this study should be entered on the study specific CRFs accurately and in a timely manner. All source documents from which CRF entries are derived should be present in the participant's medical records. During a monitoring visit, the original CRFs for each participant will be checked against source documents at the study site by the clinical site monitor.

Each CRF and DCF will consist of an original plus a copy. The copy will be forward to a designated person within the Obstetrix Research Team within 30 days of delivery and discharge of both mother and infant. The original will be kept at the site. Instances of

missing or un-interpretable data will be discussed with the investigator and study staff for resolution.

c. Access to Source Data and Monitoring. During the course of the study, a representative of Obstetrix Research team, or a designated representative [a Clinical Research Associate (CRA) or monitor] will make site visits as frequently as necessary to review protocol compliance, compare CRFs and individual participant's medical records, and ensure that the study is being conducted according to pertinent regulatory requirements. CRF entries will be verified with source documentation. The review of medical records will be performed in a manner to ensure that participant confidentiality is maintained.

It is the principal investigator's (PIs)obligation to ensure that documentation of all relevant data such as relevant medical history, date of study enrollment, visit dates, results of examinations or tests, and adverse events is correctly entered in the participant's file.

Checking of the CRFs for completeness and clarity, and cross-checking with source documents in the presence of the investigator or designee, will be required to monitor the progress of the study. Moreover, the FDA, IRBs, and/or the Sponsor's Clinical Quality Assurance group may wish to carry out such source data checks and/or on-site audit inspections. Direct access to source data will be required for these inspections and audits; they will be carried out giving due consideration to data protection and medical confidentiality. The investigator agrees to give the auditor access to all relevant documents for review. The same applies in case of an inspection by the authorities.

Obstetrix or designated representatives will affirm and uphold the principle of the subject's right to protection against the invasion of privacy. Throughout the study, all data will only be identified by participant number. Anonymity of the data will be maintained in all data analyses.

d. Archiving Study Records. According to ICH guidelines and FDA Regulations, essential documents should be retained for a minimum of 2 years after the last approval of a marketing application in an ICH region and until there are no pending or contemplated marketing applications in an ICH region or at least 2 years have elapsed since the formal discontinuation of clinical development of the investigational product. However, these documents should be retained for a longer period if required by the applicable legal requirements and/or local IRB/institutional requirement.

e. Good Clinical Practice. The procedures set out in this study protocol are designed to ensure that the Sponsor and investigator abide by the principles of the Good Clinical Practice (GCP) guidelines of the International Conference on Harmonization (ICH), and of the Declaration of Helsinki (2000, with clarification in 2004), and all applicable FDA Regulations. The study also will be carried out in keeping with local legal requirements.

f. Informed Consent. Prior to the administration of any tests or procedures required in this protocol, the investigator or designee will obtain informed consent in writing from all participants considered for entry into this study. Each participant, investigator or designee, and witness (as applicable) must personally sign and date the informed consent. Copies of the

informed consent will be retained in the medical record and the participant's study binder. A copy of the signed informed consent will be given to the participant to take home.

The consent form and as necessitated by local law, an additional participant information leaflet for study participation, will explain the nature of the study, its objectives and potential risks. Furthermore, it will detail the requirement of the participant, all trial procedures, alternative procedures and the fact that she is free to withdraw her consent at any time without reason. Details of indemnity and insurance are also stated.

As required by ICH-GCP the participant will give in writing her authorization that the study data may be given for review to the responsible authorities and that the monitor will be granted direct access to the participant's original medical records for verification of clinical data. The participant's information and ICF will be available in the participant's native language.

If a protocol amendment is required, the ICF will be revised if applicable to reflect the changes to the protocol. After the ICF is revised, it must be reviewed and approved by the appropriate IRB, and signed by all participants subsequently enrolled in the study as well as those previously enrolled in the study.

g. Protocol Approval and Amendment. Before the start of the study, the study protocol and/or other relevant documents will be approved by the IRB in accordance with local legal requirements. This protocol is to be followed exactly. To alter the protocol, amendments must

be written, receive approval from the appropriate personnel, and receive IRB approval prior to implementation (if appropriate).

Administrative changes (not affecting the participant benefit/risk ratio) may be made without the need for a formal amendment. All amendments will be distributed to all protocol recipients, with appropriate instructions.

h. Confidentiality. All study findings and documents will be regarded as confidential. The investigator and members of his/her research team must not disclose such information without prior written approval from the Sponsor.

The anonymity of participating participants must be maintained. Participants will be identified on CRFs and other documents submitted to the Sponsor by their participant number, initials and/or birth date, not by name. Documents not to be submitted to the Sponsor that identify the participant (e.g., the signed informed consent) must be maintained in confidence by the investigator.

i. Publication. At the conclusion of the study, a multi-center (if applicable) publication of the primary study outcomes will be prepared for publication in a reputable scientific journal. The analysis of other pre-specified and non pre-specified endpoints will be performed by Obstetrix Research Group's designated data management team.

j. Site Training. All investigators and appropriate study staff will be required to participate in a site training (or initiation if appropriate) to provide orientation and training regarding the applicable procedures, protocol and CRFs.

k. Required Reports. The following reports are required of the investigator:

i. Reports to the Obstetrix CRE of any SAEs immediately or at least within 24 hours of the study site becoming aware of such events

ii. Report to the Obstetrix CRE, within 72 working days, of withdrawal of approval by the reviewing IRB or regulatory authority

iii. Report to the Obstetrix CRE and the reviewing IRB of any deviation from the protocol to protect the life or physical well being of a participant in an emergency situation. Such notice will be given as soon as possible, but in no event later than 5 working days after the emergency occurred

iv. Reports of any study procedure(s) being performed without prior written informed consent being obtained. Such notice will be given to the Obstetrix CRE and the reviewing IRB within 10 working days of the study procedure(s) being performed

v. Final report submitted to the Obstetrix CRE and the reviewing IRB as soon as possible, but no later than 3 months, of the conclusion of the study or the conclusion of the investigator’s participation in the study

vi. Other reports to the reviewing IRB as may be required

3.12 Compliance with Regulations & Conventions

To protect the rights and welfare of participants, the study will be conducted in conformance with the Declaration of Helsinki, applicable FDA guidelines, the ICH E6 guidelines on Good Clinical Practice (GCP), and/or the laws and regulations of the participating country, whichever affords the greater protection to the human participant.

All investigators, sub-investigators and study personnel are required to read and follow the protocol, as well as any literature that accompanies the products prior to conducting the study procedure for the first time. All investigators participating in this study will be required to be trained in the proper use of the study procedures and in the components of this protocol by the sponsor.

The PI is ultimately responsible for the conduct of this study; however, he/she may designate a member of his/her staff to assist with the collection of data and completion of CRFs. The designee(s) will be documented on an authorization form that is signed by the PI and kept in the Regulatory Binder, to be updated as necessary.

Obtaining informed consent in accordance with national policy is mandatory for participant participation. All participant data is kept confidential and procedures will be implemented to ensure that participant confidentiality is not compromised. If alternative consent materials are used, they must be approved by Obstetrix CRE and the IRB at the clinical site prior to use. Documentation of the approved informed consent must be provided to Obstetrix CRE prior to study commencement at the clinical site.

After Informed Consent has been obtained, all screening procedures have been performed, and eligibility for the study has been confirmed, the participant will be considered enrolled in the study. After this point, the reason(s) must be documented on the CRF for any participant who withdraws, or for any reason cannot complete this study.

4. References

ACOG Committee on Obstetric Practice. Antenatal corticosteroid therapy for fetal maturation. ACOG Committee Opinion 273: 1-3, American College of Obstetricians and Gynecologists, 2002.

ACOG Committee on Obstetric Practice. Use of progesterone to reduce preterm birth. ACOG Committee Opinion 291: 1-2, American College of Obstetricians and Gynecologists, 2003.

ACOG Committee on Practice Bulletins. Premature rupture of membranes. ACOG Practice Bulletin 80: 1-13, American College of Obstetricians and Gynecologists, 2007.

Amon E, Lewis SV, Sibai BM, Villar MA, Arheart KL. Ampicillin prophylaxis in preterm premature rupture of the membranes: a prospective randomized study. Am J Obstet Gynecol 1988;159:539-43.

Ananth CV, Savitz DA, Williams MA. Placental abruption and its association with hypertension and prolonged rupture of membranes: a methodologic review and metaanalysis. Obstet Gynecol 1996;88:309-18.

Armstrong J, Nageotte M for the Society for Maternal-Fetal Medicine. Can progesterone prevent preterm birth? Contemp Obstet Gynecol 2005 (Oct);30-43.

Bengtson JM, van Marter L, Barss VA, et al. Pregnancy outcome after premature rupture of membranes at or before 26 weeks' gestation. Obstet Gynecol 1989;73:921-7.

Beydoun SN, Yasin SY. Premature rupture of the membranes before 28 weeks: conservative management. Am J Obstet Gynecol 1986;155:471-9.

Caritis SN, Rouse DJ, Peaceman AM, et al. Prevention of preterm birth in triplest using 17 alpha-hydroxyprogesterone caproate: a randomized controlled trial. Obstet Gynecol 2009; 113:285-92

Caughey AB, Robinson JN, Norwitz ER. Contemporary diagnosis and management of preterm premature rupture of membranes. Rev Obstet Gynecol 2008;1:11-22.

Combs CA, McCune M, Clark R, Fishman A. Aggressive tocolysis does not prolong pregnancy or reduce neonatal morbidity after preterm premature rupture of the membranes.

Da Fonseca EB, Bittar RE, Carvalho MH, Zugaib M. Prophylactic administration of progesterone by vaginal suppository to reduce the incidence of spontaneous preterm birth in women at increased risk: a randomized placebo-controlled double-blind study. Am J Obstet Gynecol 2003;188:419-24.

Dale PO, Tanbo T, Bendvold E, Moe N. Duration of the latency period in preterm premature rupture of the membranes. Maternal and neonatal consequences of expectant management. Eur J Obstet Gynecol Repord Biol 1989;30:257-62.

DeFranco EA, O'Brien JM, Adair CD, et al. Vaginal progesterone is associated with a decrease in risk for early preterm birth and improved neonatal outcome in women with a short cervix: a secondary analysis from a randomized, double-blind, placebo-controlled trial. Ultrasound Obstet Gynecol 2007;30:697-705.

Fonseca EB, Celik E, Parra M, et al. Progesterone and the risk of preterm birth among women with a short cervix. New Engl J Med 2007;357:462-9.

Garite TJ, Freeman RK. Chorioamnionitis in the preterm gestation. Obstet Gynecol 1982;59:539-45.

Garite TJ. Premature rupture of the membranes: the enigma of the obstetrician. Am J Obstet Gynecol 1985;151:1001-5.

Goldstein P, Berrier J, Rosen S, Sacks HS, Chalmers TC. A metaanalysis of randomized control trials of progestational agents in pregnancy. Br J Obstet Gynaecol 1989;96:265-74.

Golub MS, Kaufman FL, Campbell MA, Li L-H. Evidence on the developmental and reproductive toxicity of progesterone. Reproductive and Cancer Hazard Assessment Section, California Environmental Protection Agency, Draft Document, 2004.

Gonen R, Hannah ME, Milligan JE. Does prolonged preterm premature rupture of the membranes predispose to abruptio placentae? Obstet Gynecol 1989;74:347-50.

Gonzalez-Quintero M, Istwan NB, Rhea DJ, et al. Gestational age at initiation of 17-hydroxyprogesterone caproate (17P) and recurrent preterm delivery. J Matern Fetal Neonatal Med 2007;20:249-52.

Grable IA, Garcia PM, Perry D, Socol ML. Group B Streptococcus and preterm premature rupture of membranes: a randomized, double-blind clinical trial of antepartum ampicillin. Am J Obstet GYnecol 1996;175:1036-42.

How HY, Barton JR, Istwan NB, Rhea DJ, Stanziano GJ. Prophylaxis with 17 alpha-hydroxyprogesterone caproate for prevention of recurrent preterm delivery: does gestational age at initiation of treatment matter? Am J Obstet Gynecol 2007;197:260.e1-4.

Johnson JW, Egerman RS, Moorhead J. Cases with ruptured membranes that "reseal." Am J Obstet Gnecol 1990;163:1024-30.

Keirse MJNC. Progesterone administration in pregnancy may prevent preterm delivery. Br J Obstet Gynaecol 1990;97:149-54.

Kenyon S, Boulvain M, Neilson J. Antibiotics for preterm rupture of the membranes: a systematic review. Obstet Gynecol 2004;104:1051-7.

Martin JA, Hamilton BE, Sutton PD, et al. Births: Final data for 2005. National Vital Statistics Reports 2007;56:1-104.

McGregor JA, French JI, Seo K. Antimicrobial therapy in preterm premature rupture of membranes: results of a prospective, double-blind, placebo-controlled trial of erythromycin. Am J Obstet Gynecol 1991;165:632-40.

Meis PJ, Klebanoff M, Tom E, et al. Prevention of recurrent preterm delivery by 17 alpha-hydroxyprogesterone caproate. New Engl J Med 2003;348:2379-85.

Mercer BM. Management of premature rupture of the membranes before 26 weeks' gestation. Obstet Gynecol Clin North Am 1992;19:335-31.

Mercer BM, Miodovnik M, Thurnau GR, et al. Antibiotic therapy for reduction of infant morbidity after preterm premature rupture of the membranes: a randomized controlled trial. JAMA 1997;278:989-95.

Mercer BM, Goldenberg RL, Meis PJ, et al. The Preterm Prediction Study: Prediction of preterm premature rupture of membranes through clinical findings and ancillary testing. Am J Obstet Gynecol 2000;183:738-45.

NIH Consensus Development Panel. Antenatal corticosteroids revisited: repeat courses. Obstet Gynecol 2001;98:144-50).

O'Brien JM, Adair CD, Luis DF, et al. Progesterone vaginal gel for the reduction of recurrent preterm birth: primary results from a randomize double-blind, placebo-controlled trial. Ultrasound Obstet Gynecol 2007;30:687-96.

Oates-Whitehead R, Haas D, Carrier J. Progestogen for preventing miscarriage (Cochrane Review). In the Cochrane Library, John Wiley & Sons, Ltd., Chichester, UK, 2004.

Porreco RP, Heyborne KD, Shapiro H. Amniocentesis in the management of preterm premature rupture of the membranes: a retrospective cohort analysis. J Matern Fetal Neonatal Med 2008;21:573-9

Rebarber A, Ferrara LA, Hanley ML, et al. Increased recurrence of preterm delivery with early cessation of 17-alpha-hydroxyprogesterone caproate. Am J Obstet Gynecol 2007;196:224.e1-4.

Romero R. Prevention of spontaneous preterm birth: the role of sonographic cervical length in identifying patients who may benefit from progesterone treatment. Ultrasound Obstet Gynecol 2007;30:675-86.

Rouse DJ, Caritis SN, Peaceman AM, et al. A trial of 17 alpha-hydroxyprogesterone caproate to prevent prematurity in twins. New Engl J Med 2007;357:454-61.

Simpson GF, Harbert GM. Use of betamethasone in management of preterm gestation with premature rupture of membranes. Obstet Gynecol 1985;66:168-75.

Tita AT, Rouse DJ. Progesterone for preterm birth prevention: an evolving intervention. Am J Obstet Gynecol 2009;200:219-24.

Wright LL, Verter J, Younes N, et al. Antenatal corticosteroid administration and neonatal outcome in very low birth weight infants: the NICH Neonatal Research Network. Am J Obstet Gynecol 1995;173:269-74.
