# Supplementary material for: 17-Hydroxyprogesterone caproate to prolong pregnancy after preterm rupture of the membranes: early termination of a double-blind, randomized clinical trial
Source: BMC Res Notes. 2011 Dec 29;4:568. doi: 10.1186/1756-0500-4-568 (PMC3260323; doi:10.1186/1756-0500-4-568)
Supplement: Additional file 2 — Data, 17P for PROM; Obstetrix Medical Group data, 17-hydroxyprogesterone caproate for preterm rupture of membranes; Results table, de-identified raw data for each participant. [file 1756-0500-4-568-S2.PDF]

Obstetrix Medical Group Data
17-hydroxyprogesterone caproate for preterm rupture of membranes

study code	hospital_ code	Group Assignment group 1=17P 2=placebo	GA at PROM	GA at Randomi zation	Days from PROM to enroll	GA at 1st 17P dose	GA Delivery	Primary Outcome (34 w or FLM>32)	Maternal Age	Pre pregnancy weight (preferred) or if prepregnan cy weight is unknown - earliest recorded weight in pregnancy	BMI	Parity	Number of previous preterm deliveries?
8103	COGS	1	31.00	31.14	1	31.14	33.57	N	30.28	142	20.97	0	0
8252	PRSL	1	30.14	30.71	4	30.71	32.00	N	29.95	142	20.97	0	0
8311	GSAM	1	15.29	26.57	79	26.57	29.00	N	29.03	135	20.52	0	0
8341	DSAM	1	23.71	24.00	2	24.00	24.29	N	24.01	210	33.89	2	0
8101	COGS	2	22.43	23.00	4	23.00	27.86	N	42.18	135	21.79	1	0
8102	COGS	2	28.14	28.14	0	28.14	28.57	N	35.59	111	17.91	0	0
8131	STLH	2	19.86	24.00	29	24.00	25.14	N	29.66	178	27.06	0	0
8191	SWED	2	29.57	29.71	1	29.71	30.29	N	25.36	135	24.69	0	0
8251	PRSL	2	29.86	30.14	2	30.14	30.43	N	38.14	135	22.46	0	0
8281	SMED	2	31.57	31.71	1	31.71	33.86	N	34.40	190	30.66	1	0
8312	GSAM	2	24.43	25.29	6	25.29	25.43	N	24.42	212	37.55	1	0
8313	GSAM	2	24.29	24.43	1	24.43	25.29	N	36.50	198	31.95	0	0

Obstetrix Medical Group Data
17-hydroxyprogesterone caproate for preterm rupture of membranes

study code	Marital Status?	Highest level of education completed (check one)	Race / Ethnicity	Hx of smoking during pregnancy	Hx of ETOH use during pregnancy	Amphet/ Cocaine or Heroin	Marijuana	Was patient transported from another Hospital?	How was pregnancy conceived? (select one)	If Received progesterone supplementation during pregnancy, what was the GA in wks and days
8103	Married/Living with partner	College Graduate	White	No	None	No	No	No	IVF	9W 0 D
8252	Married/Living with partner	Unknown	White	No	None	No	No	Yes	Spontaneous	N/A
8311	Single/Widowed	Unknown	Black or Afr	No	None	No	Yes	No	Spontaneous	N/A
8341	Married/Living with partner	Unknown	White	Unknown	None	No	No	No	Spontaneous	NULL
8101	Married/Living with partner	College Graduate	White	No	None	No	No	No	IUI	12 WEEK 0 DA
8102	Married/Living with partner	College Graduate	White	No	None	No	No	No	Spontaneous	NULL
8131	Married/Living with partner	College Graduate	White	No	None	No	No	No	Spontaneous	NA
8191	Married/Living with partner	Unknown	White	No	None	No	No	No	Spontaneous	first trimester-d
8251	Married/Living with partner	College Graduate	White	No	None	No	No	Yes	Spontaneous	NULL
8281	Married/Living with partner	Unknown	White	Yes	None	No	No	Yes	Spontaneous	NULL
8312	Single/Widowed	Unknown	American Ir	Yes	Rare (1-3 X	No	Yes	Yes	Spontaneous	N/A
8313	Married/Living with partner	College Graduate	Black or Afr	No	None	No	No	Yes	Spontaneous	N/A

Obstetrix Medical Group Data
17-hydroxyprogesterone caproate for preterm rupture of membranes

study code	Anti-hypertensive used during pregnancy ?	Insulin used during pregnancy?	MgS04 given IV within 12 hours of delivery?	ACS Before PROM, After PROM, Incomplete or NONE	IF YES, #1 Tocolytic used during the first 48 hours following administration of the first dose of ACS.	Were Tocolytics used AFTER the first 48 hours following administration of first dose of ACS?	IF Yes, Antibiotic #1 used AFTER ROM but prior to Labor.	IF Yes, Antibiotic #2 used after ROM but prior to Labor	IF Yes, Antibiotic #3 used after ROM but prior to Labor	Were any other antibiotics used AFTER ROM (not captured above)	Were antibiotics given PROPHYLACTICALLY DURING labor and delivery/Perioperative
8103	No	No	No	After	Nifedipine (	No	Ampicillin	Zithromycin	Amoxicillin	No	Yes
8252	No	No	No	After	Terbutaline	No	Ampicillin	Zithromycin	Zithromycin	No	Yes
8311	Yes	Yes	No	After	Not Given	Yes	Other - see comm	Other - see c	Zithromycin	Yes - see c	No
8341	No	No	No	Before	MgS04	Yes	Zithromycin	Gentamycin	Ancef	No	Yes
8101	No	No	No	After	Not Given	Yes	Amoxicillin	Zithromycin	Not Given	No	Yes
8102	No	No	Yes	After	MgS04	No	Zithromycin	Ampicillin	Amoxicillin	Yes - see c	Yes
8131	No	No	No	After	Not Given	No	Amoxicillin	Zithromycin	Flagyl	No	No
8191	No	No	Yes	After	Not Given	No	Ampicillin	Zithromycin	Ampicillin	No	Yes
8251	No	No	Yes	After	MgS04	No	Ampicillin	Zithromycin	Not Given	No	No
8281	No	No	No	After	MgS04	No	Ancef	Zithromycin	Zithromycin	Yes - see c	Yes
8312	No	Yes	No	After	MgS04	Yes	Ampicillin	Amoxicillin	Zithromycin	No	No
8313	Yes	No	Yes	After	MgS04	No	Ampicillin	Amoxicillin	Not Given	No	Yes

Obstetrix Medical Group Data
17-hydroxyprogesterone caproate for preterm rupture of membranes

study code	If Yes, #1 Antibiotic given prophylactically during Labor and Delivery/Perioperative	If Yes, #2 Antibiotic given prophylactically during Labor and Delivery/Perioperative	Number of doses	Wks from randomization to delivery (latency)	Latency (days) randomization to delivery	Rupture of membranes confirmed by what method (#1)?	Rupture of membranes confirmed by what method (#2)?	Rupture of membranes confirmed by what method (#3)?
8103	Ancef	Not Given	2	2.43	17.0	Nitrazine Test	Gross Pooling	N/A
8252	Cephalospo	Not Given	2	1.29	9.0	Gross Pooling	U/S exam shows oligohydr	N/A
8311	Not Given	Not Given	3	2.43	17.0	U/S exam shows oligohyd	N/A	N/A
8341	Ancef	Gentamycir	1	0.29	2.0	Amnisure	U/S exam shows oligohydr	Gross Pooling
8101	Ancef	Not Given	5	4.86	34.0	Amnisure	N/A	N/A
8102	Ampicillin	Not Given	1	0.43	3.0	Gross Pooling	Amnisure	U/S exam shows ol
8131	Not Given	Not Given	2	1.14	8.0	U/S exam shows oligohyd	N/A	N/A
8191	Ampicillin	Amoxicillin	1	0.57	4.0	Nitrazine Test	Gross Pooling	Positive Fern Test
8251	Not Given	Not Given	1	0.29	2.0	U/S exam shows oligohyd	Gross Pooling	N/A
8281	Ancef	Not Given	3	2.14	15.0	Gross Pooling	Nitrazine Test	Positive Fern Test
8312	Not Given	Not Given	1	0.14	1.0	Nitrazine Test	Gross Pooling	Positive Fern Test
8313	Ampicillin	Not Given	1	0.86	6.0	Amnisure	U/S exam shows oligohydr	N/A

Obstetrix Medical Group Data
17-hydroxyprogesterone caproate for preterm rupture of membranes

study code	Amniotic Fluid specimen was obtained for Fetal Lung Maturity (FLM) by the following way	What test was used to determine Fetal Lung Maturity (FLM)	Fetal Lung Maturity Results	Gender / Sex of infant	Birth weight (in grams)	APGARS @ 5 minutes	Short Reason for Delivery
8103	By Vaginal collection	Lamellar Bodies	Not Mature	Male	1570	9	Fetal
8252	Not done	Not Done	Not Done	Female	1890	9	Fet+Infx
8311	Not done	Not Done	Not Done	Male	1240	6	Fetal
8341	Not done	Not Done	Not Done	Male	610	8	Spont
8101	Not done	Not Done	Not Done	Male	770	6	Fetal
8102	Not done	Not Done	Not Done	Male	1280	8	Spont
8131	Not done	Not Done	Not Done	Male	1000	7	Spont
8191	By Amniocentesis	Fluorescense pol	Not Mature	Male	1660	8	Spont
8251	Not done	Not Done	Not Done	Male	1540	9	Spont
8281	By Amniocentesis	L/S	Not Mature	Female	2307	7	Infect
8312	Not done	Not Done	Not Done	Male	885	9	Fet+Matrn
8313	Not done	Not Done	Not Done	Male	865	6	Infect

Obstetrix Medical Group Data
17-hydroxyprogesterone caproate for preterm rupture of membranes

study code	Primary Reason for delivery	Secondary reason for delivery	Route of Delivery
8103	Delivery initiated d/t known or suspected fetal jeopardy	No Secondary Indication	Primary C/S
8252	Delivery initiated d/t known or suspected fetal jeopardy	Delivery initiated d/t known or suspected infection	Primary C/S
8311	Delivery initiated d/t known or suspected fetal jeopardy	No Secondary Indication	Primary C/S
8341	Spontaneous Labor	Delivery initiated d/t known or suspected infection	SVD
8101	Delivery initiated d/t known or suspected fetal jeopardy	No Secondary Indication	Repeat C/S
8102	Spontaneous Labor	No Secondary Indication	SVD
8131	Spontaneous Labor	Unknown	Primary C/S
8191	Spontaneous Labor	No Secondary Indication	SVD
8251	Spontaneous Labor	No Secondary Indication	SVD
8281	Delivery initiated d/t known or suspected infected	No Secondary Indication	Primary C/S
8312	Delivery initiated d/t known or suspected fetal jeopardy	Delivery initiated d/t worsening maternal condition	Repeat C/S
8313	Delivery initiated d/t known or suspected infected	No Secondary Indication	Primary C/S

Obstetrix Medical Group Data
17-hydroxyprogesterone caproate for preterm rupture of membranes

study code	Primary reason for operative intervention	NICU Days	Newborn hosp days	Respiratory distress syndrome (RDS)	Bronchopulmonary dysplasia (BPD)	Intraventricular hemorrhage (IVH)	Periventricular leukomalacia (PVL)	Proven sepsis within first 72 hrs of Life	Retinopathy of prematurity
8103	Fetal Distress (NRFHR)	23	23	No	No	No IVH	No	No	No eye exam done
8252	Fetal Distress (NRFHR)	51	51	Yes	No	Grade 4 (severe)	No	No	No ROP noted on exam
8311	Malpresentation	70	70	Yes	Yes	Grade 1-2	No	No	No ROP noted on exam
8341	No Operative Intervention	24	24	Yes	No	Grade 3 (severe)	No	No	No eye exam done
8101	Malpresentation	148	148	Yes	Yes	No IVH	No	No	NULL
8102	No Operative Intervention	46	46	Yes	No	No IVH	No	No	Stage 1-2 ROP
8131	Malpresentation	0	0	Yes	No	Unknown	No	No	No eye exam done
8191	No Operative Intervention	43	43	Yes	No	No IVH	No	No	No ROP noted on exam
8251	No Operative Intervention	41	41	Yes	No	No IVH	No	No	No ROP noted on exam
8281	Malpresentation	6	9	No	No	No IVH	No	No	No eye exam done
8312	Abruption	84	84	Yes	Yes	Grade 1-2	No	No	No ROP noted on exam
8313	Fetal Distress (NRFHR)	83	83	Yes	Yes	Grade 1-2	No	No	Stage 1-2 ROP

Obstetrix Medical Group Data
17-hydroxyprogesterone caproate for preterm rupture of membranes

study code	Necrotizing enterocolitis (NEC)	Neonatal death	Comp Neo Morbid	Abnormal ities	Pneum onia	Clinical Chorioamni onitis with this pregnancy?	Maternal Sepsis?	Diabetes with this pregnancy?	Pre-Eclampsia with this pregnancy?
8103	No NEC	No	N	No	No	No	No	None	None
8252	No NEC	No	Y	No	No	No	No	None	None
8311	No NEC	No	Y	Yes	No	No	No	Type 1, DX prior to pregnancy	None
8341	Yes - NEC Present	Yes	Y	No	No	Yes	No	None	None
8101	No NEC	No	Y	Yes	No	No	No	Gestational, TX with diet only	None
8102	No NEC	No	Y	Yes	No	No	No	None	Mild Preeclampsia
8131	No NEC	Yes	Y	No	No	No	No	None	None
8191	No NEC	No	Y	Yes	No	Yes	No	None	None
8251	No NEC	No	Y	No	No	No	No	None	None
8281	No NEC	No	N	No	No	No	No	None	None
8312	No NEC	No	Y	Yes	No	No	No	Gestational, TX with medication	None
8313	No NEC	No	Y	No	No	No	No	None	None
